# Group 2 innate lymphoid cells are key in lipid transfer protein allergy pathogenesis

**DOI:** 10.3389/fimmu.2024.1385101

**Published:** 2024-04-25

**Authors:** Francisca Palomares, Natalia Pérez-Sánchez, Nazaret Nieto, Rafael Núñez, José Antonio Cañas, María del Carmen Martín-Astorga, Anyith Cruz-Amaya, María José Torres, Ibon Eguíluz-Gracia, Cristobalina Mayorga, Francisca Gómez

**Affiliations:** ^1^ Allergy Research Group, Instituto de Investigación Biomédica de Málaga-IBIMA Plataforma Andalusian Centre for Nanomedicine and Biotechnology (BIONAND), Málaga, Spain; ^2^ Allergy Unit, Hospital Regional Universitario de Malaga, Málaga, Spain; ^3^ Medicine Department, Universidad de Málaga-UMA, Málaga, Spain

**Keywords:** food allergy, lipid transfers proteins, innate lymphoid cells, antigen-presenting cells, type 2 immune response, Th2-cells

## Abstract

**Background:**

Immunopathology in food allergy is characterized by an uncontrolled type 2 immune response and specific-IgE production. Recent studies have determined that group 2 innate lymphoid cells (ILC2) participate in the food allergy pathogenic mechanism and their severity. Our objective was to investigate the role of ILC2 in peach-allergic patients due to non-specific lipid transfer protein (Pru p 3) sensitization.

**Methods:**

The immune response in peripheral blood mononuclear cells was characterized in lipid transfer protein-allergic patients and healthy controls. We have analyzed the Pru p 3 uptake on ILC2, the expression of costimulatory molecules, and their involvement on the T-cell proliferative response and cytokine production under different experimental conditions: cytokines involved in group 2 innate lymphoid cell activation (IL-33 and IL-25), Pru p 3 as main food allergen, and the combination of both components (IL-33/IL-25+Pru p 3) using cell sorting, EliSpot, flow cytometry, and confocal microscopy.

**Results:**

Our results show that Pru p 3 allergen is taken up by group 2 innate lymphoid cells, regulating their costimulatory molecule expression (CD83 and HLA-DR) depending on the presence of Pru p 3 and its combination with IL-33/IL-25. The Pru p 3-stimulated ILC2 induced specific GATA3^+^Th2 proliferation and cytokine (IL-4, IL-5, and IL-13) production in lipid transfer protein-allergic patients in a cell contact-dependent manner with no changes in Tbet^+^Th1- and FOXP3^+^Treg cell differentiation.

**Conclusions:**

The results indicate that in lipid transfer protein-allergic patients, the responsible allergen, Pru p 3, interacts with group 2 innate lymphoid cells, promoting a Th2 cell response. Our results might be of interest *in vivo*, as they show a role of group 2 innate lymphoid cells as antigen-presenting cells, contributing to the development of food allergy. Consequently, group 2 innate lymphoid cells may be considered as potential therapeutic targets.

## 1 Introduction

Food allergy (FA), one of the most frequent entities in allergy consultations whose prevalence has increased by 50% during the last decade, affects 5% of the adult population and 8% of children (1, 2). Food allergy can be severe depending on different factors as co-sensitizations or a genetic predisposition to allergic reactions (3).

Foods of plant origin are the most common triggers in both adults and adolescents (2), with non-specific lipid transfer proteins (nsLTP) being the main allergens involved. nsLTP are highly stable, not modified during thermal and digestion processing (4, 5), which might explain their allergenicity and its involvement in severe systemic symptoms (6, 7). These proteins are panallergens, and consequently, although some patients can react to a single nsLTP (mainly Pru p 3, from peach), patients are frequently sensitized to nsLTP from different allergenic sources, thus hampering clinical management (6, 8).

Classical mechanisms in FA need the involvement of the innate immune system with antigen-presenting cells (APCs), like dendritic cells (DCs), recognizing, processing, and presenting the food allergen to T-cells (9), which differentiate toward IL-4-, IL-5-, and IL-13-producing Th2 cells (10). This is accompanied by specific immunoglobulin E (sIgE) production by B-cell-derived plasma cells and T regulatory (Treg) response blockage (11). However, knowledge about the underlying mechanisms involving other innate system elements in FA is limited.

Recent studies raised a possible role of innate lymphoid cells (ILC) in driving inflammatory diseases, mainly searching biomarkers and/or therapeutic targets (12, 13). ILC are involved in immunity, homeostasis regulation, and inflammation; therefore, an alteration in its levels may lead to the development of allergic reactions (14–16). ILC are grouped into three main subtypes defined by the combination of surface markers, transcription factors, and cytokine production (TGFβ and IFNγ for ILC1; GATA3 and IL-4, IL-5, IL-9 and IL-13 for ILC2; and RORγt and IL-22 for ILC3) (17). Although ILC2 have been associated with allergic pathologies (18) by inducing a faster and antigen-independent response upon activation, their implication in FA has not been completely elucidated, with many studies being performed only in experimental FA models (19). These studies describe that, in the presence of the antigen, epithelial cells produce IL-25 and IL-33 and thymic stromal lymphopoietin that activate and increase ILC2 survival, respectively. Activated ILC2 promote Th2 cells and IgE-producing B-cells, blocking Treg cells, to induce the anaphylactic reaction (20, 21). Moreover, IgE-mediated activation of mast cells induce IL-33 secretion, activating and amplifying the ILC2-mediated response (22). In humans, in FA context, our group recently reported evidence of the presence of circulating ILC2 producing IL-13 and IL-4 related to LTP-allergic patients (LTP-AP). We showed the ILC2 involvement in type 2 (T2) immune response, showing a correlation between Th2 cells and reaction severity (23).

Recently, it has been described that ILC2 express MHCII and costimulatory molecules (CD80 and CD86), showing their possible participation as APCs, and interact with T-cells to promote Th2 polarization in inflammatory pathologies (24–26). Related to PD-L1, despite being a regulatory marker observed in LTP-AP treated with specific allergen immunotherapy (27), its expression in ILC2 cells, both *in vitro* and *in vivo*, could be associated with a Th2 response or with the expression of GATA3 and the production of IL-13 as demonstrated in infectious pathologies (*Nippostrongylus brasiliensis*) (28). These studies suggest that further analyses are needed to explain the implication and functioning of ILC2 on T-cell polarization and FA.

Here we show that ILC2 can take up Pru p 3 and express different costimulatory molecules that will affect the presentation to T-cells *in vitro*. Indeed the Pru p 3-preactivated ILC2 induced the differentiation to a Th2 phenotype with a lack in Tbet^+^Th1/FOXP3^+^Treg activity. This interaction between ILC2 and T-cells is essential in FA regulation.

## 2 Materials and methods

### 2.1 LTP-allergic patients and tolerant controls

LTP-AP with a confirmed diagnosis of peach allergy was determined by a positive skin prick test (SPT), sIgE to peach LTP (Pru p 3), or a positive double-blind placebo-controlled food challenge (DBPCFC) to unpeeled peach when in doubt of the diagnosis. DBPCFC was not performed in patients with more than two episodes of anaphylaxis after peach ingestion in the 2 years preceding the study. Severity was classified according to FASS-5 (29). A tolerant group of subjects (HC) presenting negative SPT and Pru p 3-sIgE and with confirmed tolerance to unpeeled peach was included (**Table 1**). LTP-AP and HC were evaluated in relation to the presence of respiratory allergic diseases (allergic rhinitis), according to ARIA classification (**Table 1**), and all samples were obtained out of the seasonal period. The study was conducted at the Allergy Service and Research Laboratory of the Hospital Regional Universitario de Málaga-Instituto de Biomedicina de Málaga (HRUM-IBIMA), Spain, in accordance with the Declaration of Helsinki and was approved by the local ethics committee (code: 2201-N-20). All participants signed informed consent forms.

**Table 1:** Clinical characteristics and allergological workup results.

N^o^.	S	Age	Pollen/mite sensitization	Rhinitis	Rhinitis severity (ARIA)	A/AD	sIgE (kU/L)	SPT (mm^2^)	Reaction oFASS-5
LTP-AP1	**F**	45	Yes (grass and olive)	Yes	Intermitent-mild	No	1.45	Pos	Grade 2
LTP-AP2	M	42	No	Yes	Intermitent-mild	No	1.82	Pos	Grade 2
LTP-AP3	F	36	Yes (Der p 1)	Yes	Intermitent-mild	No	2.29	Pos	Grade 2
LTP-AP4	F	29	Yes (*D. pteronyssinus*)	Yes	Intermitent-mild	No	12,7	Pos	Grade 2
LTP-AP5	F	24	Yes (Olive)	Yes	Intermitent-mild	No	15.8	Pos	Grade 3
LTP-AP6	F	32	No	Yes	Persistent-mild	No	77	ND	Grade 2
LTP-AP7	F	37	Yes (Timothy and Plantanus)	Yes	Persistent-mild	No	2.66	Pos	Grade 2
LTP-AP8	F	21	Yes (*D. pteronyssinus*, Ole e 1 and 7)	No	NA	No	21.7	Pos	Grade 3
LTP-AP9	F	40	No	No	NA	No	8.67	Pos	Grade 3
LTP-AP10	M	18	Yes (Art v 3 and Pla a 3)	No	NA	No	4.5	Pos	Grade 4
LTP-AP11	M	57	No	No	NA	No	47.7	Pos	Grade 4
LTP-AP12	F	44	Yes *(D. pteronyssinus)*	Yes	Persistent-mild	No	1.34	Pos	Grade 4
HC-1	F	40	No	No	No	No	<0.35	–	T
HC-2	M	30	Yes (Grass)	Yes	Persistent‐mild	No	<0.35	–	T
HC-3	F	39	No	No	No	No	<0.35	–	T
HC-4	M	29	Yes (Grass)	Yes	Persistent‐mild	No	<0.35	–	T
HC-5	F	38	No	No	No	No	<0.35	–	T
HC-6	M	55	Yes (HDM)	Yes	Persistent‐mild	No	<0.35	–	T
HC-7	F	27	No	No	No	No	<0.35	–	T
HC-8	M	53	Yes (Olive)	Yes	Persistent‐mild	No	<0.35	–	T
HC-9	F	39	No	No	No	No	<0.35	–	T
HC-10	M	25	No	No	No	No	<0.35	–	T
HC-11	F	40	No	No	No	No	<0.35	–	T
HC-12	F	24	No	No	No	No	<0.35	–	T

N^o^., number of subjects; S, sex; HDM, house dust mite; LTP-AP, lipid transfer protein-allergic patients; SPT, skin prick test (specific IgE to Pru p 3); T, tolerant; HC, tolerant control; A/AD, asthma/atopic dermatitis; Pos, positive; NA, Not Available; ND, Not Detected; - indicates a negative SPT.

### 2.2 Skin prick tests

SPT were performed according to European guidelines (30) using extracts of aeroallergens (olive tree, *Phleum*, *Artemisia*, plane tree, and *D. pteronyssinus*), plant foods related to LTP (peanut, hazelnut, walnut, almond, tomato, and lettuce), and peach LTP (Pru p 3-enriched) (all from ALK-Abelló). A wheal size >3 mm^2^ compared to the one induced by a negative saline control was considered positive.

### 2.3 Double-blind placebo-controlled food challenge

DBPCFC to unpeeled peach was performed out of the pollen season (olive grass pollen and plane tree mainly, to avoid confusion with respiratory symptoms due to pollen symptoms) following European guidelines (30, 31).

### 2.4 Pru p 3-specific immunoglobulin (Ig) determination

Serum sIgE to Pru p 3 was determined using ImmunoCAP (Thermo Fisher Scientific, Uppsala, Sweden) following the manufacturer’s recommendations. Results higher than 0.35 kU/L were considered positive.

### 2.5 Sample collection and storing

Peripheral blood was obtained by venopunction. Peripheral blood mononuclear cells (PBMCs) were isolated using Ficoll gradient and stored in liquid nitrogen. The PBMCs and serum were managed at the biobank according to the standard procedures of HRUM-IBIMA Biobank, Andalusian Public Health System Biobank.

### 2.6 Cell sorting and flow cytometry

ILC2 (Linage^-^CD123^-^CD15^-^CD161^+^CD127^+^CRTH2^+^) (23) and T-cells (Linage^+^ CD123^+^CD15^+^CD3^+^CD4^+^) were isolated from PBMCs by using cell sorting (MoFloAstrios, Beckman Coulter, Brea, CA, USA) with purity >95% using fluorochromes-labeled-monoclonal antibodies (mAb) [Becton Dickinson Company (BD), Biolegend and MiltenyiBiotec (Bergen NJ, San Diego, CA, USA, and Germany, respectively]. We display the details of fluorochrome-conjugated monoclonal antibodies (moAbs) in **Supplementary Table S1**. Linage included CD3, CD14, CD16, CD19, CD20, and CD56 markers. The reanalysis by flow cytometry of the ILC2 after sorting, shown in **Supplementary Figure S1**, ensured the purity of the population. LIVE/DEAD fixable near-IR dead cell stain kit (Thermo Fisher Scientific) was used to determine cell viability. The results after sorting are presented as percentages in LTP-AP and HC.

ILC2 and T-cell absolute number was determined using the CountBright Plus kit (Thermo Fisher Scientific) and evaluated with a MoFloAstrios flow cytometer. The absolute cell count was obtained by multiplying the number of positive ILC2 and T-cell events by the number of positive bead events and dividing by the expected number of bead events (**Supplementary Figures S1–S3**).

### 2.7 Confocal microscopy

The sorted ILC2 and T-cells were fixed in PBS containing 1% paraformaldehyde, washed with PBS, stored, and protected from light until analysis. Sub-membrane actin and nuclei (DNA) were labeled with Alexa Fluor 488 Phalloidin (Sigma-Aldrich, Munich, Germany) and Hoechst 33258 (Sigma-Aldrich), respectively (32). The cells were either transferred to optical-bottom 12-well plates (Thermo Fisher Scientific) in PBS for observation by confocal microscopy (32). The samples were analyzed using a Leica DM6000 inverted microscope connected to a Leica SP5 laser scanning confocal system and Fiji software to determine the cell integrity after sorting.

### 2.8 Pru p 3 uptake by confocal microscopy and flow cytometry

For Pru p 3 internalization analysis in ILC2 by confocal microscopy and flow cytometry, natural Pru p 3 (ROXAL Oststeinbek, Germany) was labeled with Alexa Fluor 647 NHS Ester (succinimidyl ester) (Pru p 3-AF647) according to the manufacturer’s instructions (Thermo Fisher Scientific). After incubation with constant stirring in the dark, the uncoupled free AF647 was removed by gel filtration column (PD MiniTRap G-25 column, GE Healthcare, Chicago, IL, USA) with PBS. Pru p 3-AF647 was quantified with a Nanodrop 2000 spectrophotometer (Thermo Fisher Scientific).

A mean of 1,000 ILC2 was cultured with 25 µg/mL Pru p 3-AF647 (27) with/without the cocktail cytokines, IL-25/IL-33 (CK) both at 50 ng/mL (33) (R&D Systems) at 48 h and 37°C, as previously described for Pru p 3 uptake by DCs, as APC (32). Afterward, ILC2 was fixed and labeled with Alexa Fluor 488 Phalloidin and Hoechst 33258, as previously described (32). For flow cytometry, ILC2 was acquired in a FACSCanto II Cytometer (BD), and data was analyzed using FlowJo software (BD). The results were expressed as mean fluorescence intensity (MFI) on ILC2.

### 2.9 *In vitro* activation assay in ILC2

The sorted ILC2 were cultured in 96-well plates (Thermo Fisher Scientific) with different experimental conditions; cocktail cytokines, Pru p 3 (25 µg/mL) (27), and CK+Pru p 3 for 48 h at 37°C in 5% CO_2_. Unstimulated ILC2 in complete medium was considered as the negative control. Changes in CD83, CD86, HLA-DR, and PD-L1 molecule (BD) expression were analyzed using MoFloAstrios (Beckmand-Coulter). We display the details of fluorochrome-conjugated moAbs in **Supplementary Table S1**. Data were analyzed using FlowJo software (BD). The unstimulated cells were considered controls for flow cytometric analyses, following the gate strategy shown in **Supplementary Figure S2**. The results were expressed as percentage expression for each marker.

### 2.10 ELISpot assay in ILC2

After sorting, ILC2 (an average of 250 cells per experimental condition) were cultured. The number of IL-13- and IL-4-secreting ILC2 after different stimulations, cocktail cytokines, Pru p 3, and CK+Pru p 3, was measured by ELISpot assay (Human IL-13 and IL-4 ELISpot Basic Kits, Mabtech, Nacka Strand, Sweden). These cytokines were chosen as cytokines of inflammatory response by ILC2, as we have previously described (23). ELISpot Multiscreen HTS plates (Millipore, Darmstadt, Germany) were coated overnight with IL-13 and IL-4 monoclonal antibodies, and the protocol followed the manufacturer’s recommendations. The medium and unstimulated ILC2 were considered as negative controls. The number of IL-13- and IL-4-secreting cells was determined using an ELISpot Bioreader (R) 6000 (BioSys, Karben, Germany). The results were expressed as number of spots of cytokines producing ILC2.

### 2.11 Co-cultures and T lymphocyte proliferative response

Sorted T-cells (**Supplementary Figure S3**) were labeled with 5,6-carboxyfluorescein diacetate N-succinimidyl ester (CFSE, Thermo Fisher Scientific) and cultured alone or in the presence of ILC2 in a 1:100 ratio of ILC2/T-cells at a final volume of 250 μL in different experimental combinations, cocktail cytokines, Pru p 3, and CK+Pru p 3 and without stimulus for 7 days at 37°C and 5% CO_2_. The same co-cultures were performed using 3-μm Transwell porous plates at a final volume of 600 μL. For all culture assays, on day 7, T-cells were recovered and phenotyped as CD3^+^CD4^+^ (T-cells), CD3^+^CD4^+^CRTH2^+^GATA3^+^ (GATA3^+^Th2 cells), following the strategies shown in **Supplementary Figure S4**, CD3^+^CD4^+^Tbet^+^ (Tbet^+^Th1-cells), and CD3^+^CD4^+^CD127^-^CD25^+^FOXP3^+^ (FOXP3^+^Treg cells), following the strategies shown in **Supplementary Figure S5**, in MoFloAstrios. We display the details of fluorochrome-conjugated moAbs in **Supplementary Table S1**. Nuclear transcription factors were evaluated by human FOXP3 buffer (BD). Proliferation was determined by the percentage of CFSE_low_ cells. The results were presented as percentages of CFSE_low_ for each T-cell subpopulation.

### 2.12 Cytokine determination

Cytokine production (IL-4, IL-5, IL-9, IL-10, IL-13, IFNγ, and IL-27) from supernatant cell cultures (T-cells alone, ILC2-T-cells contact or with Transwell) collected after 7 days was determined with a human ProcartaPlex Multiplex Immunoassays kit (Thermo Fisher Scientific), following the manufacturer’s indications, and analyzed in Bio-Plex 200 using Bio-Plex Data Analysis Software (Bio-Rad, Hercules, CA, USA). The results were presented as concentration (pg/mL) of each cytokine.

### 2.13 Statistical analysis

Data were analyzed using Shapiro–Wilk test to determine the normal distribution, but most variables were fitted to non-parametric distribution. We compared the effects of the different experimental conditions (unstimulated cells, cocktail cytokines, Pru p 3, and CK+Pru p 3) between the different groups of subjects (LTP-AP vs. HC) using Kruskal–Wallis test. If this test showed significant differences between groups, then we applied Mann–Whitney *U*-test to compare the LTP-AP and HC groups. This showed four *post hoc* tests. Moreover, the comparisons between related samples in the same group (LTP-AP or HC) were carried out using Friedman test. If this test showed significant differences, Wilcoxon test was applied, showing six *post hoc* tests. The correlations were carried out using Spearman tests. The significant differences were reported with an asterisk, representing a *p*-value <0.05. The statistical analysis was carried out using GraphPad Prism7.

## 3 Results

### 3.1 Clinical characteristics of LTP-allergic patients and tolerant controls

A total of 12 LTP-AP, 75% female patients with a mean age 35.5 years, and 12 sex- and age-matched HC were included. In the LTP-AP group, six (50%) patients presented a reaction after unpeeled peach ingestion as grade 2, three (25%) as grade 3, and three (25%) as grade 4. Sensitization to Pru p 3 was confirmed with a positive SPT to peach and/or Pru p 3-sIgE (median of 6.59 ± 23.5 kU/L).

### 3.2 Pru p 3 uptake by activated ILC2

**Figure 1:**
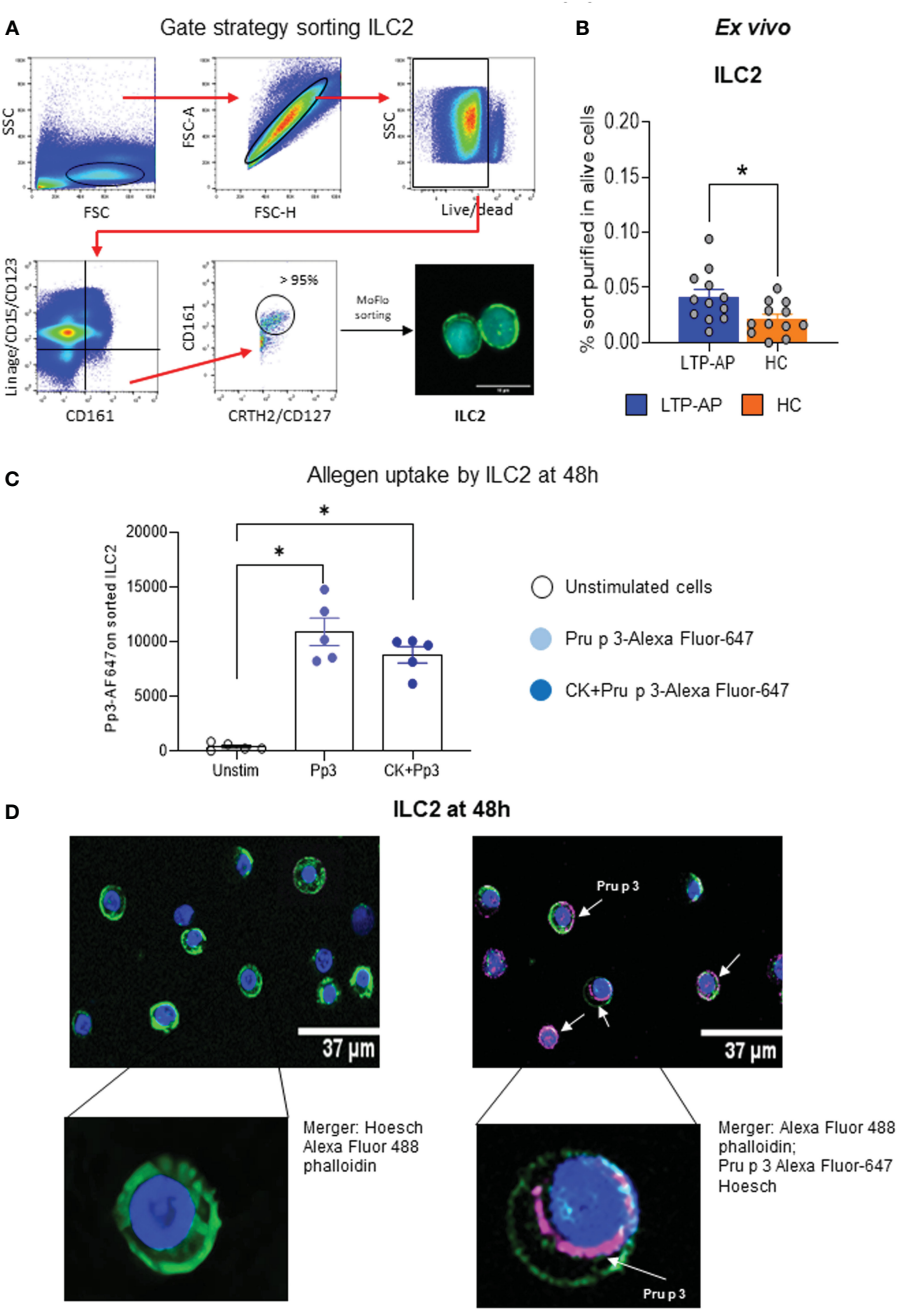
**(A)** Gating strategy for sorting ILC2, and ILC2 confocal images after sorting. **(B)** Frequency of sorted ILC2 on alive PBMCs in LTP-AP and HC (*N* = 12 for both groups). The bars with symbols represent the mean and SEM of ILC2 frequency. Mann–Whitney test for pairwise comparisons in unrelated samples. **(C)** Fluorescence-labeled Pru p 3 (Pp3) uptakes by ILC2 under different experimental conditions in LTP-AP (Pp3-Alexa Fluor-647 at 25 µm/mL and cocktail cytokines: IL-25 and IL-33 at 50 ng/mL). The bars with symbols represent the mean and SEM of mean fluorescence intensity. Friedman test, followed by Wilcoxon test, was used to detect differences. Significant *p*-values are marked with an asterisk (*p* < 0.05). **(D)** Representative confocal images of ILC2 incubated with Pp3 at 25 µg/mL in LTP-AP. The confocal images show different cellular regions. The sub-membrane actin was stained with Atto 488-phalloidin (green), the nuclei with Hoechst (blue), and Pp 3 with Alexa Fluor 647 (pink).

ILC2 purification from PBMCs by flow cytometry and confocal microscopy morphology are shown in **Figure 1A**. The data indicate that purified ILC2 percentages were significantly higher in LTP-AP compared to HC with high viability in both groups (**Figure 1B**; **Supplementary Figure S1**), confirming that the sorting protocol was optimum to ILC2. The analysis of Pru p 3 uptake capacity of ILC2 under different stimulation conditions, using Pru p 3-AF647 in flow cytometry, indicated that this preferentially happens in ILC2 independently of the presence of cocktail cytokines in LTP-AP (**Figure 1C**) and HC (data not shown). The confocal microscopy images confirmed this internalization of the Pru p 3 surrounding the nucleus after 48 h (**Figure 1D**).

### 3.3 Pru p 3 upregulates the costimulatory marker and produces T2 cytokines on ILC2

**Figure 2:**
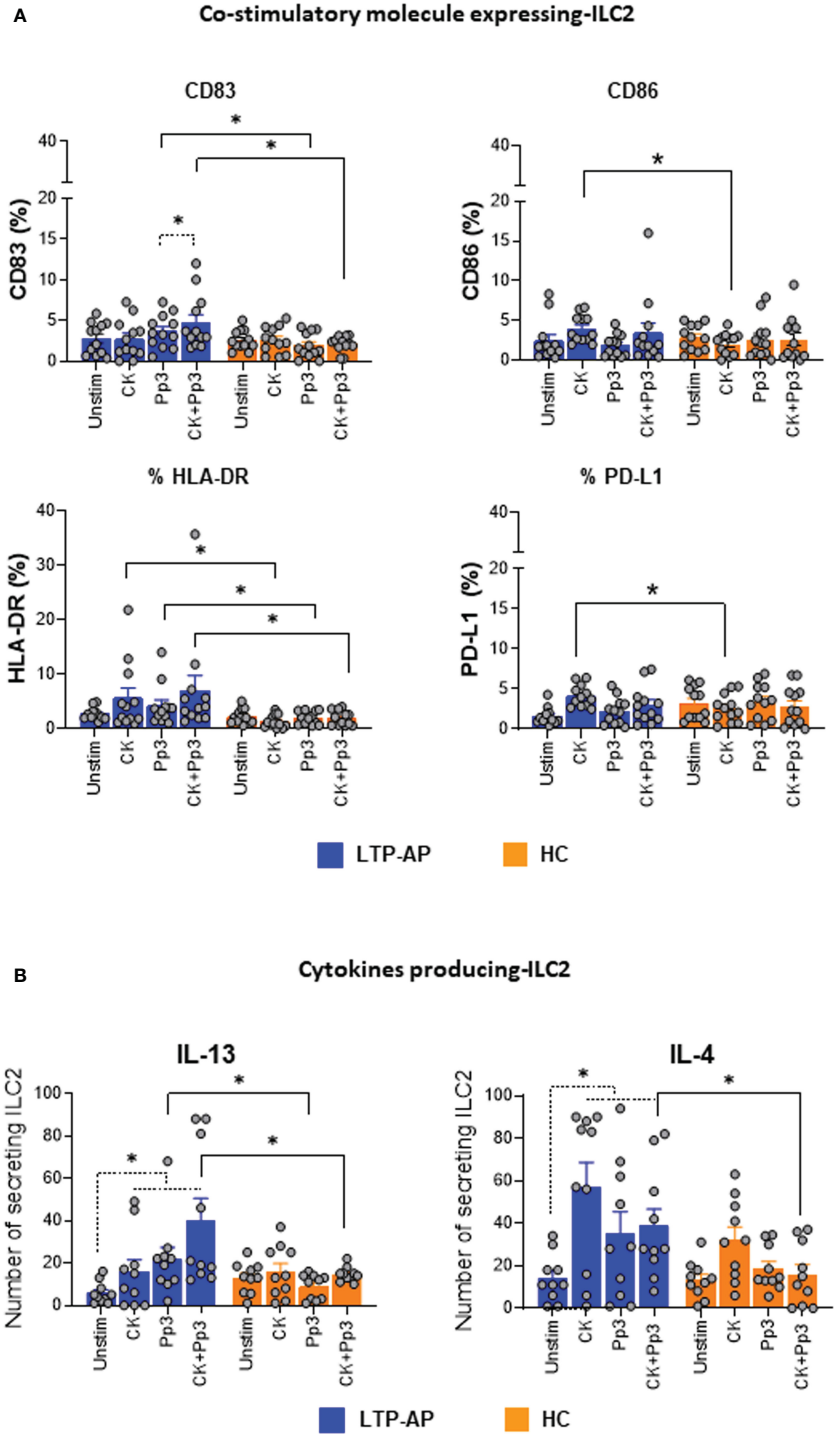
**(A)** Co-stimulatory molecule expression on ILC2. Percentages of ILC2 expressing for the different surface markers under different experimental conditions. Unstimulated cells (Unstim), cocktail cytokines (IL-25 and IL-33) at 50 ng/mL, Pru p 3 (Pp3) allergen (at 25 µm/mL), and CK+Pp3 for LTP-AP (*N* = 12) and HC (*N* = 12). **(B)** Cytokine producing ILC2. Number of cytokine-secreting ILC2 under different experimental conditions in LTP-AP and HC (*N* = 10 in both groups). The bars with symbols represent the mean and SEM of ILC2 number for **(A, B)**. Kruskal–Wallis test, followed by Mann–Whitney test, was used for detecting differences between unrelated groups (continuous line). Friedman test, followed by Wilcoxon test, was used for pairwise comparisons in related samples (dotted line). The dashed horizontal line indicates significant differences when comparing unstimulated cells vs. the rest of the experimental conditions (CK, Pp3, and CK+Pp3) in both types of cytokines in LTP-AP. Significant *p*-values are marked with an asterisk (*p* < 0.05).

We evaluated the changes of co-stimulatory molecules (CD83 and CD86), HLA-DR, and PD-L1 expression on ILC2 after Pru p 3 stimulation with/without cocktail cytokines (**Figure 2A**). Cocktail cytokines induced a significant upregulation of CD86, HLA-DR, and PD-L1 on ILC2 from LTP-AP compared to HC, independently of the presence of Pru p 3. Interestingly, CD83 and HLA-DR significantly increased their expressions in LTP-AP compared to HC under Pru p 3 and CK+Pru p 3 stimulation. This effect was amplified by the combination with cocktail cytokines, being significant for CD83 compared to Pru p 3 alone in LTP-AP (**Figure 2A**). No differences in the regulation of these markers were observed in HC for any experimental condition.

The ability of ILC2 to produce IL-4 and IL-13 in response to stimulation with cocktail cytokines, Pru p 3, and CK+Pru p 3 was investigated by ELISpot (**Figure 2B**). A significantly higher number of IL-13-secreting ILC2 was found in LTP-AP compared to HC when stimulating with Pru p 3, independently of the presence of cocktail cytokines, whereas IL-4-secreting ILC2 cells were only significantly increased when stimulated with the combination CK+Pru p 3 (**Figure 2B**). In the case of HC, Pru p 3 stimulation seems to reduce IL-13- and IL-4-producing cells compared to cocktail cytokines.

In LTP-AP, ILC2 increased the IL-13 and IL-4 production under the different stimuli compared to unstimulated cells, which suggested that these ILC2, in the presence of Pru p 3, could increase their activity.

### 3.4 Pru p 3-stimulated-ILC2 promotes Th2 cell proliferative response in LTP-AP

We sought to determine the effect of ILC2 stimulated with cocktail cytokines, Pru p 3, and their combination on CD3^+^CD4^+^T-cell responses. Isolated T-cell morphology, frequency, and absolute numbers are shown in **Supplementary Figure S3** and were similar in LTP-AP and HC with a viability after sorting close to 100% in both groups.

**Figure 3:**
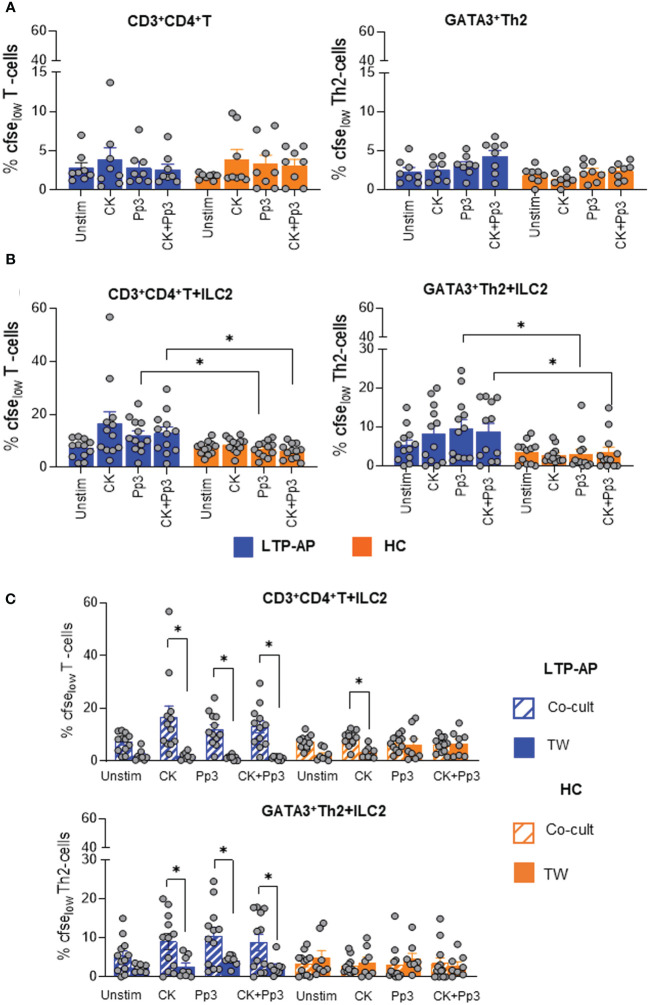
The bars with symbols represent the mean and SEM of percentages of CFSE_low_ expression **(A)** in T-cell cultures alone for LTP-AP and HC (*N* = 8 in both groups), **(B)** in ILC2/T-cell co-cultures (LTP-AP and HC *N* = 12 in both groups), and **(C)** in ILC2/T-cell co-cultures using Transwell plate for LTP-AP and HC (*N* = 8, respectively) under different experimental conditions. Unstimulated cells (Unstim), cocktail cytokines (IL-25 and IL-33) at 50 ng/mL, Pru p 3 (Pp3) allergen at 25 µm/mL, and CK+Pp3. Kruskal–Wallis test, followed by Mann–Whitney test, was used to detect differences in between unrelated groups (continuous line). Friedman test, followed by Wilcoxon test, was used for pairwise comparisons in related samples (dotted line). Significant *p*-values are marked with an asterisk (*p* < 0.05).

Cell cultures of CD3^+^CD4^+^T-cell (alone) under the different conditions, cocktail cytokines, Pru p 3, and their combination, did not show cell proliferation in any study group, probably due to APCs’ absence (**Figure 3A**). Under co-culture conditions, Pru p 3- and CK+Pru p 3-stimulated ILC2 promoted a significant proliferation of T-cells and GATA3^+^Th2 cells in LTP-AP compared to HC (**Figure 3B**). However, the presence of stimulated ILC2 in co-cultures did not induce Tbet^+^Th1 or FOXP3^+^Treg proliferation in any group (**Supplementary Figure S6**). Moreover, these proliferative responses were abolished in the presence of Transwell plates (**Figure 3C**), indicating the contact-dependent mechanism. In HC, ILC2 were capable of inducing CD3^+^CD4^+^T-cell proliferation under cocktail cytokine stimulation, and this response was inhibited using Transwell plates (**Figure 3C**). This suggests that the cocktail of cytokines induces unspecific activation of ILC2 in HC, leading to the proliferation of CD3^+^CD4^+^T-cells, but not those with a Th2 profile.

**Figure 4:**
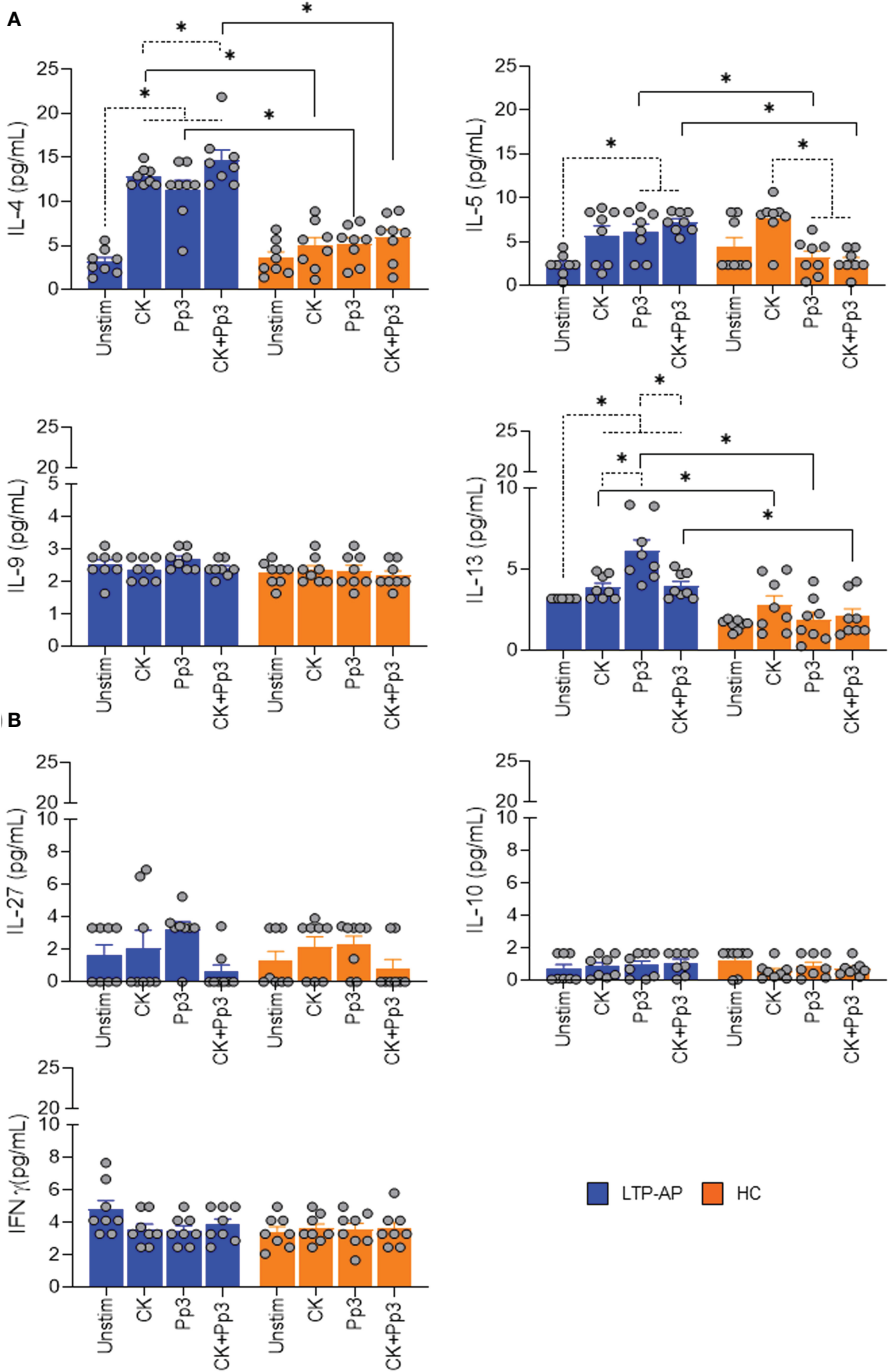
Cytokine production on cell co-culture. The bars with symbols represent the mean and SEM of the cytokine concentrations (pg/mL) in ILC2/T-cell co-cultures under different experimental conditions for LTP-AP and HC (*N* = 8 for both groups). Unstimulated cells (Unstim), cocktail cytokines (IL-25 and IL-33) at 50 ng/mL. Pru p 3 (Pp3) at 25 µm/mL, and CK+Pp3. **(A)** IL-4, IL-5, IL-9, and IL-13 production and **(B)** IL-27, IL-10, and IFNγ production on cell co-culture. Kruskal–Wallis test, followed by Mann–Whitney test, was used to detect differences in between unrelated groups (continuous line). Friedman test, followed by Wilcoxon test, was used for pairwise comparisons in related samples (dotted line). The dashed horizontal line indicates significant differences when comparing unstimulated cells vs. the rest of the experimental conditions (CK, Pp3, and CK+Pp3) in IL-4 and IL-13, and for IL-5 it indicates differences when comparing unstimulated cells vs. Pp3 and Ck+Pp3 in LTP-AP. In the case of HC, this dashed horizontal line shows significant differences between CK vs. Pp3 and CK vs. CK+Pp3. Significant *p*-values are marked with an asterisk (*p* < 0.05).

We next sought to identify the secreted cytokines in supernatant from stimulated-ILC2 and CD3^+^CD4^+^T-cell co-cultures associated with type 2 (IL-4, IL-5, IL-9, and IL-13), type 1 (IFNγ), and regulatory (IL-10 and IL-27) immune response. IL-4, IL-5, and IL-13 levels, linked to Th2 cell polarization, were significantly detected in LTP-AP compared to HC under stimulation of Pru p 3 and CK+Pru p 3, with a clear production of these cytokines from ILC2/T-cell co-cultures in the presence of food allergens (**Figure 4A**). However, no changes in IL-9 levels were detected.

Nevertheless, in LTP-AP, the IL-4, IL-5, and IL-13 levels significantly increased in the presence of different experimental conditions with respect to unstimulated cells, and this increase was significant for IL-13 levels in the presence of Pru p 3 vs. cocktail cytokines and CK+Pru p 3. Moreover, for HC, we only observed an increase in IL-5 production under CK vs. CK+Pru p 3 and vs. Pru p 3 (**Figure 4A**). The IL-27, IL-10, and IFNγ levels were not detected in co-culture supernatants, confirming the ability of ILC2 to enhance CD3^+^CD4^+^T-cell type 2 cytokine production in the presence of Pru p 3 and block the regulatory response (**Figure 4B**).

### 3.5 IL-13^+^ILC2 are increased in severe clinical phenotypes in LTP-AP

**Figure 5:**
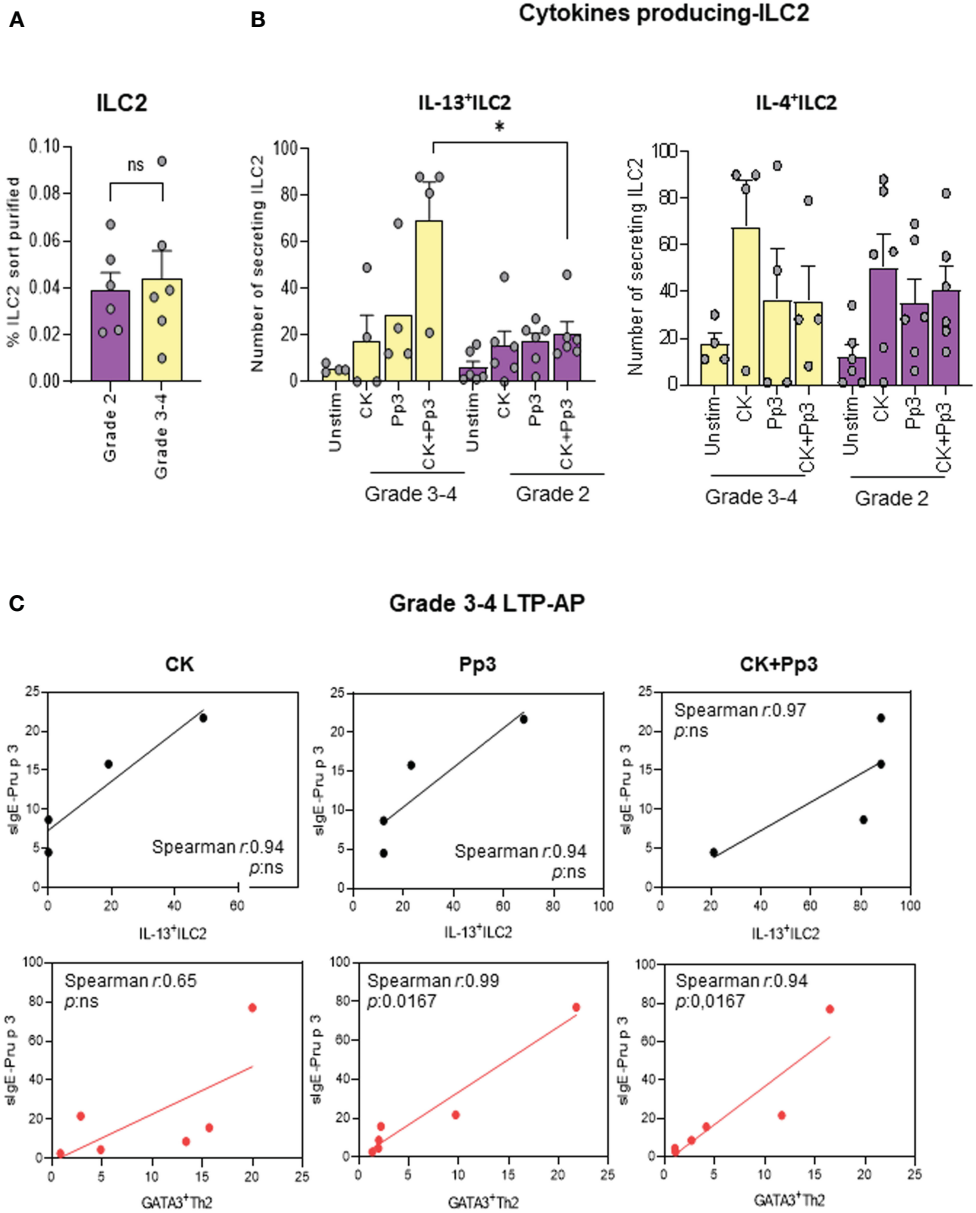
The bars with symbols represent the mean and SEM of **(A)** the percentages of purified ILC2 in grades 3 and 4 (anaphylactic) and grade 2 (urticaria) in LTP-AP (*N* = 6 for both phenotypes). Mann–Whitney test was used to detect differences in between unrelated groups. **(B)** Number of cytokines secreting ILC2 in anaphylactic (*N* = 4) and urticaria (*N* = 6) in LTP-AP. Kruskal–Wallis test, followed by Mann–Whitney test, was used to detect differences in between unrelated groups. Significant *p*-values are marked with an asterisk (*p* < 0.05). **(C)** Spearman correlation of IL13^+^ILC2 (black round symbols) and GATA3^+^Th2 cells (red round symbols) vs. Pru p 3 (Pp3) -sIgE in grades 3 and 4 LTP-AP. Significant *p*-values when *p <*0.05.

After confirming the role of ILC2 in LTP allergy, we wanted to analyze their association with reaction severity [anaphylaxis (grades 3 and 4) and urticaria (grade 2) symptoms]. A slight increase in ILC2 frequency (*ex vivo*) between clinical phenotypes was found (**Figure 5A**), being significant in IL-13-producing ILC2 in grades 3 and 4 LTP-AP compared to grade 2 LTP-AP, under stimulation with CK+Pru p 3. No differences between clinical phenotypes were found for IL-4-producing ILC2 (**Figure 5B**). However, there were positive relationships between the numbers of IL-13-producing ILC2 and GATA3^+^Th2 cells in grades 3 and 4 LTP-AP and their sIgE serum levels, being significant for sIgE-Pru p 3 vs. GATA3^+^Th2 in the presence of Pru p 3 and CK+Pru p 3 (**Figure 5C**).

## 4 Discussion

FA is an abnormal immune response to a specific food that can be sIgE-mediated (immediate hypersensitivity reactions), non-IgE-mediated (eosinophilic esophagitis) (34), and mixed (immune reactions mediated by IgE and cells) (35). Classically, DCs are considered APCs, responsible for T2 immune response in respiratory and FA pathologies (9, 36). Evidence indicates that ILC2 are implicated in the immune response of allergic diseases (36), and specifically we demonstrated that also in FA to nsLTPs (23). Although ILC2 were classically claimed to activate in an antigen-independent manner (37), we have demonstrated that they can interact with Pru p 3 food allergen and activate, increasing the expression of costimulatory molecules and the production of T2 cytokines in LTP-AP. This study, in which ILC2 demonstrate the induction of an allergen-specific immunological response in LTP-AP, confirms our previous results (23).

Recent work has shown that ILC2, under stimulation of IL-33 and IL-25 (cocktail cytokines), enhance the T2 immunity (14). However, various cell surface molecules mediating cell–cell interactions serve as important ILC2 regulators and play critical roles in ILC2-driven allergic inflammation (26). Our results confirm that, in response to cocktail cytokines, ILC2 upregulated CD86 and PD-L1, as observed in ILC2 from IL-33-treated mice and humans (38) in infectious diseases (39), leading to an increase in the effector response (40). Interestingly, this happens only in LTP-AP but not in HC, suggesting a predisposition of ILC2 to present a faster and greater reactivity, indicating an inflammatory background in allergic subjects.

In this study, we demonstrated that ILC2 also expressed the costimulatory molecule CD83 and HLA-DR under stimulation of the involved allergen Pru p 3 alone or combined with cytokines, Pru p 3+CK, with a capacity to produce T2 cytokines, mainly IL-13, in a specific way since this only happens in LTP-AP. These results showed a more efficient immunological response when both allergen and cytokines interact with ILC2. Moreover, the expression of HLA-DR and CD83 on ILC2 could be the basis of an agonist effect necessary for the activation of T-cells, as described for MHC-II and CD86 (26), and contribute to T2 immune response in FA (41).

DCs are considered the professional APCs, responsible for T2 immune response in FA, as demonstrated in LTP-AP (9, 27). In fact, the Pru p 3 allergen was able to activate DCs, increasing the expression percentages of HLA-DR, CD83, and PD-L1 in LTP-AP compared with HC inducing an increase in Th2 cell proliferation and a decrease in regulatory T-cells (reduction in IL-10 production by them) (9, 27). In line with these results, we here show that ILC2 also increase their HLA-DR expression in the presence of Pru p 3, suggesting that they may also act as APCs. Indeed ILC2 take up Pru p 3, increasing CD83 and HLA-DR expression in LTP-AP. This agrees with another study in human *in vitro* assay where ILC2 process and present Der p 1 allergen to T-cells and exacerbate a T2 inflammatory response (26). Additionally, the role of ILC2 as APCs can be supported by the fact that, in the presence of ILC2, isolated T-cells proliferate in an antigen-specific manner and by a direct cell–cell contact (ILC2/T-cells), as shown in other *in vivo* and *in vitro* studies (42, 43). Given that, our results reveal a significant proliferation of T-cells and Th2 cells in LTP-AP, particularly when there is a direct contact between ILC2/T-cells in the presence of Pru p 3 allergen and their combinations with cocktail cytokines. This specific proliferation is further validated by the absence of proliferation of both cell types in co-cultures using Transwell plates. Importantly, this proliferation of T-cell and Th2 cells is accompanied by an increased production of T2 cytokines such as IL-4, IL-5, and IL-13, which further supports the importance of the physical interaction between ILC2 and T-cells in exacerbating the food allergic response.

Afterwards, we tried to dissect the effect of ILC2 mediated by Pru p 3 allergen and their combinations with cocktail cytokines in the immunological response in LTP-AP and observed that allergen-stimulated ILC2 promote an effector response *in vitro* that was accompanied by an absence of Tbet^+^Th1- and FOXP3^+^Treg cell proliferation with suppression of IL-10, IL-27, and IFNγ production, confirming the lack of regulatory response in the allergic reaction (44). It is tempting to speculate that this lack of regulatory response is possibly blocked by IL4- or IL13-producing ILC2 as demonstrated by others (21). Regarding this, the lack of regulatory response occurs in the interaction between ILC2 and T-cells, which blocks the generation of allergen-specific Treg cells and promotes FA.

We previously showed that, depending on the clinical phenotype, there was an increase in the frequency of IL-4^+^ILC2 and IL-13^+^ILC2 in LTP-AP with grades 3 and 4 compared to grade 2 (23). Despite the limited sample size in the different clinical phenotypes, our results confirm these previous data indicating that, in the presence of Pru p 3, ILC2 can produce higher levels of IL-13 in anaphylactic LTP-AP than in urticaria. This IL-13 production was significantly higher than IL-4 production in anaphylactic LTP-AP. Furthermore, IL-13^+^ILC2 in grades 3 and 4 LTP-AP had a positive correlation with the parameters of the effector response, Pru p 3-sIgE levels, and Th2 cell proliferation. These observations, together with the link between ILC2 and the IgE-mediated adaptive response, represent an important advance in the role of ILC2 in FA (20), suggesting them as key components and a potential therapeutic target for FA in clinical practice. In fact, ILC2 activation by Pru p 3 will trigger the release of inflammatory cytokines and amplify the allergic response.

Although our study has limitations such as the small sample size or the lack of *in vitro* studies relating the possible sentinel function of DCs on ILC2 for the generation of a Th2 cell response or the blocking MHCII/T-cell receptors (TCR) interactions, it shows important advances regarding the immunological mechanism mediated by ILC2 in LTP-AP. This study focuses on studying the role of ILC2 in the effector immunological response in food allergy. Nevertheless, it would be interesting to advance in future research on the role played by these ILC2 in the sensitization phases of the response by analyzing naive T-cells.

In summary, ILC2 are involved in the immunopathology of FA acting as antigen-presenting cells and promoting the T2 effector response in LTP-AP. Our results may be of interest *in vivo*, as they show that ILC2 can develop FA, mainly in severe clinical phenotypes, and therefore could be considered as a potential therapeutic target both in clinical practice and for the development of new treatments in FA.

## Data availability statement

The datasets presented in this study can be found in online repositories. The names of the repository/repositories and accession number(s) can be found in the article/**Supplementary Material**.

## Ethics statement

The study was conducted at the Allergy Service and Research Laboratory of the Hospital Regional Universitario de Málaga-Instituto de Biomedicina de Málaga (HRUM-IBIMA), Spain, in accordance with the Declaration of Helsinki and approved by the local ethics committee include: (code: 2201-N-20). All participants signed informed consent forms. The studies were conducted in accordance with the local legislation and institutional requirements. The participants provided their written informed consent to participate in this study.

## Author contributions

FP: Conceptualization, Data curation, Formal analysis, Funding acquisition, Investigation, Methodology, Supervision, Writing – original draft, Writing – review & editing. NP-S: Conceptualization, Funding acquisition, Investigation, Writing – review & editing. NN: Formal analysis, Investigation, Methodology, Writing – review & editing. RN: Data curation, Formal analysis, Software, Writing – original draft, Writing – review & editing. JC: Validation, Writing – original draft. MM-A: Methodology, Writing – original draft. AC-A: Methodology, Writing – original draft. MT: Funding acquisition, Project administration, Resources, Supervision, Writing – review & editing. IE-G: Conceptualization, Supervision, Writing – original draft, Writing – review & editing. CM: Conceptualization, Funding acquisition, Investigation, Resources, Supervision, Validation, Writing – original draft, Writing – review & editing. FG: Conceptualization, Data curation, Funding acquisition, Investigation, Project administration, Supervision, Validation, Writing – original draft, Writing – review & editing.
