# Supplementary figures and images for: Group 2 innate lymphoid cells are key in lipid transfer protein allergy pathogenesis
Source: Front Immunol. 2024 Apr 25;15:1385101. doi: 10.3389/fimmu.2024.1385101 (PMC11079275; doi:10.3389/fimmu.2024.1385101)

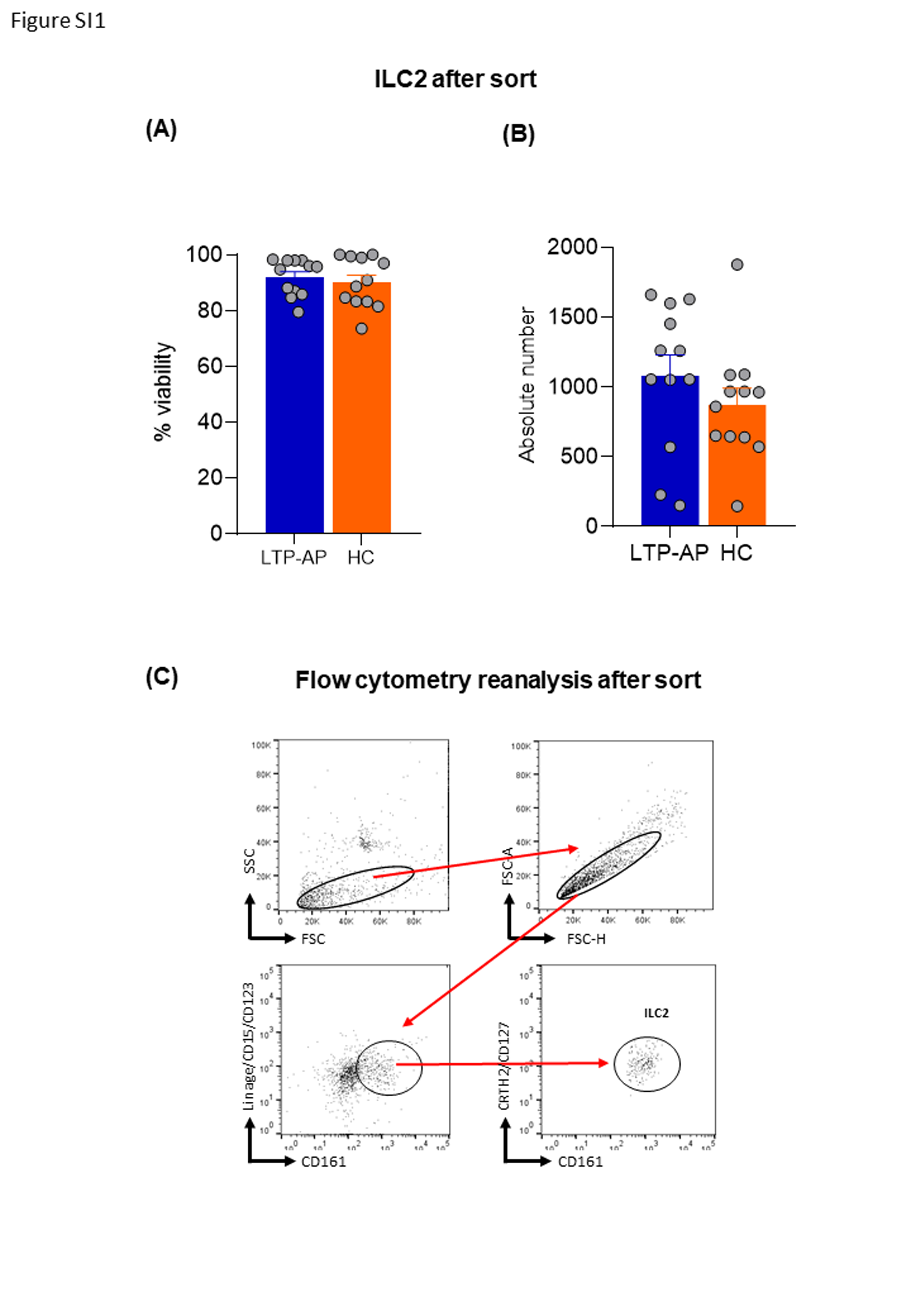

Supplement: Supplementary file 1 [file Image_1.tif]

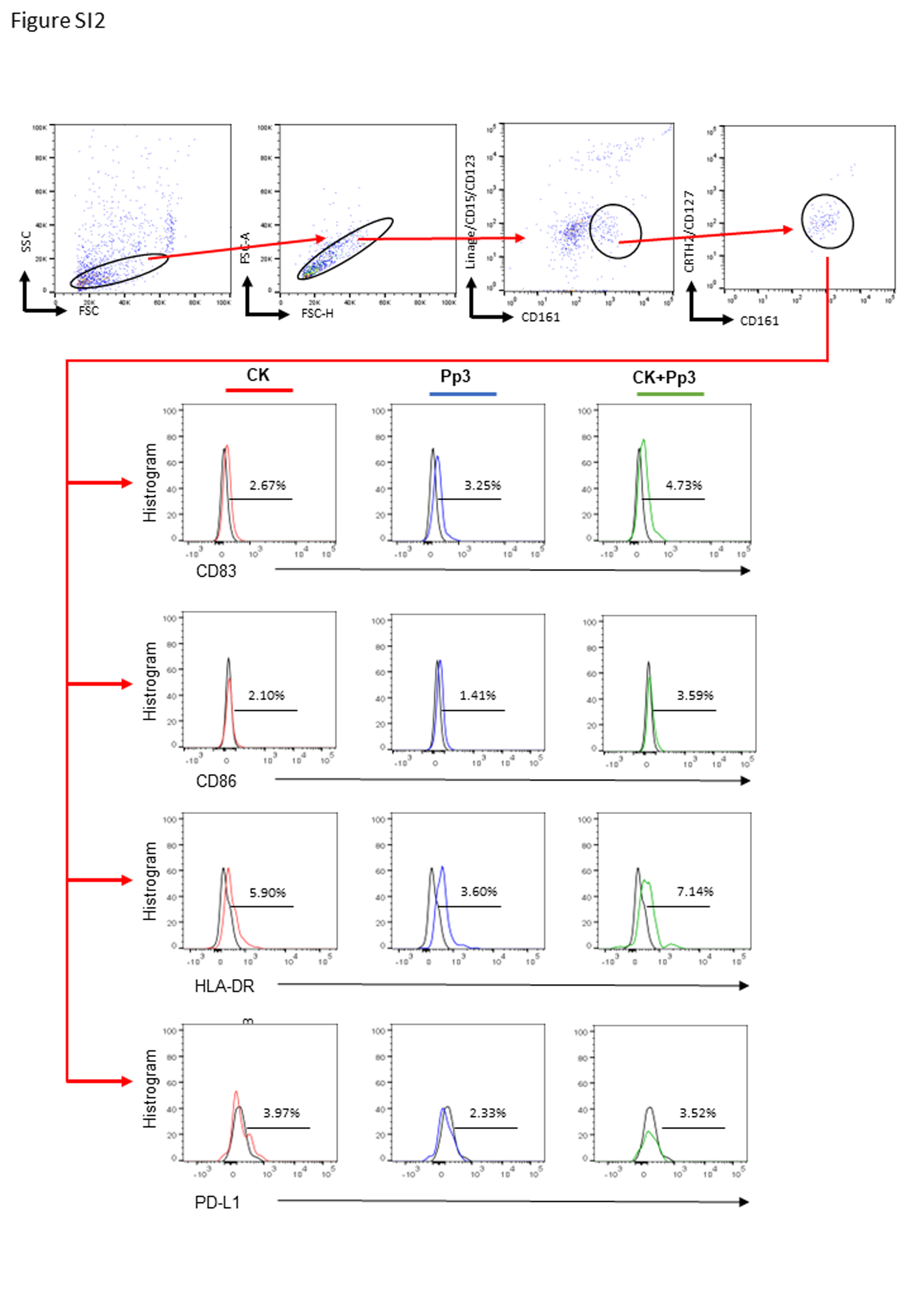

Supplement: Supplementary file 2 [file Image_2.tif]

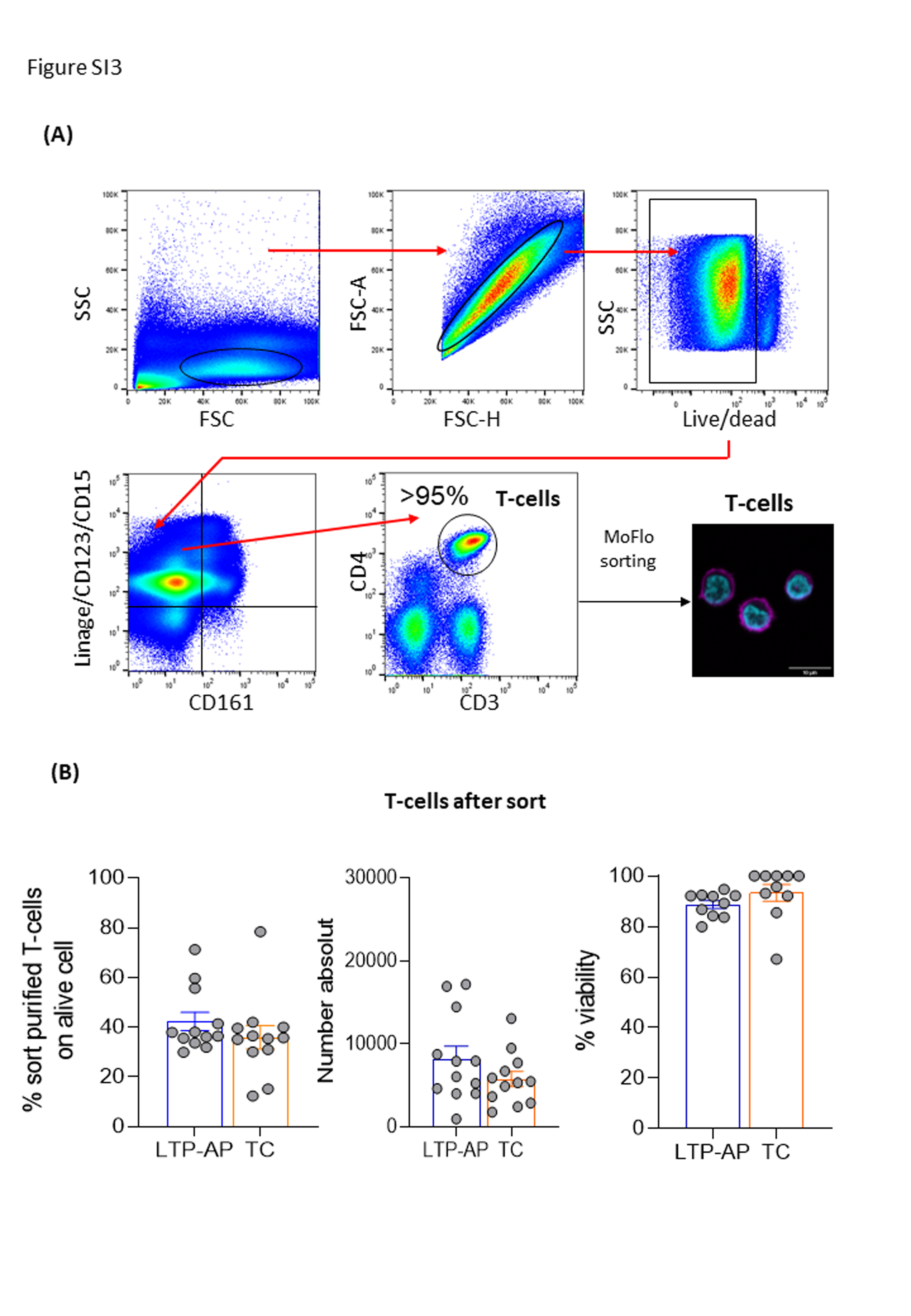

Supplement: Supplementary file 3 [file Image_3.tif]

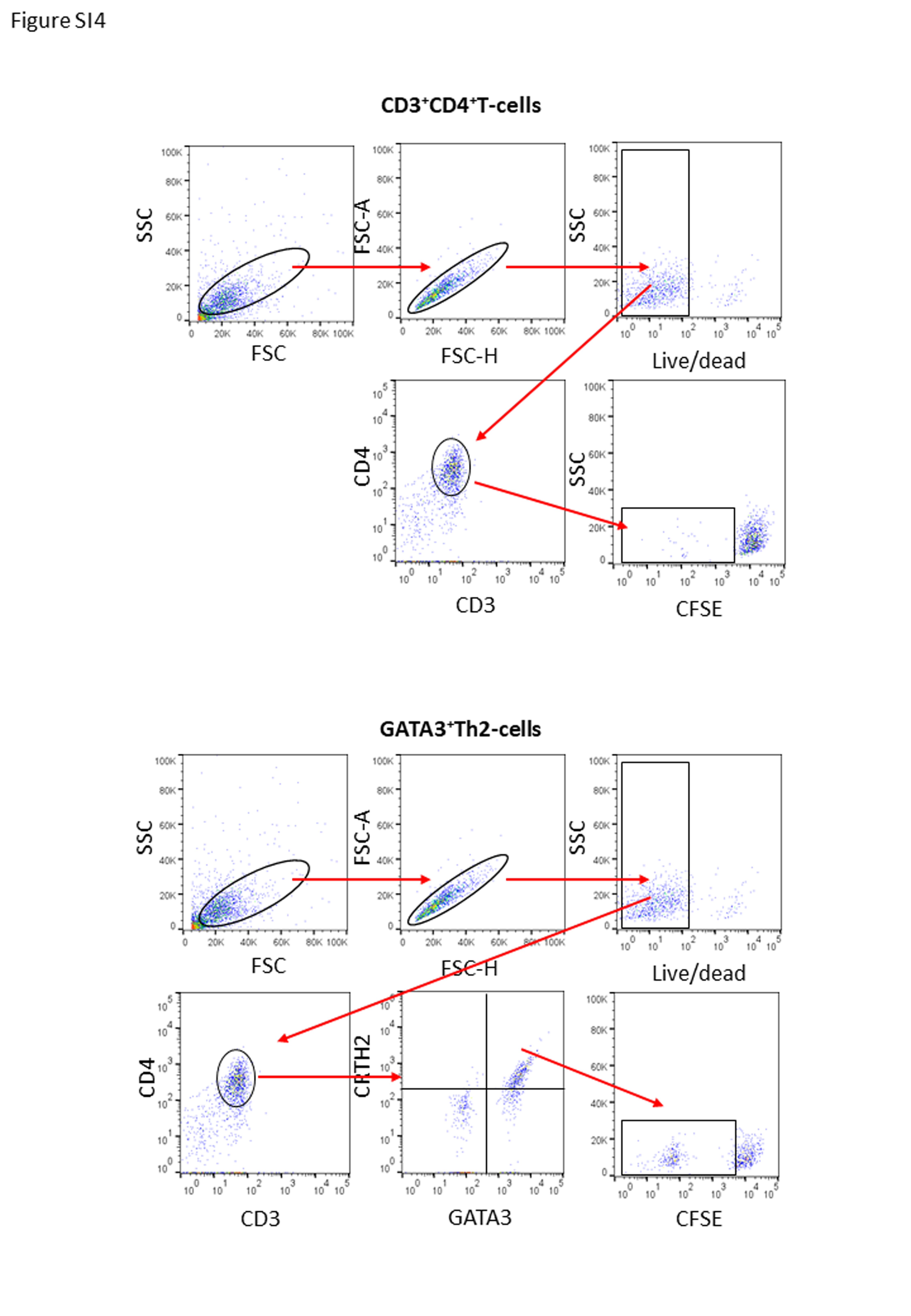

Supplement: Supplementary file 4 [file Image_4.tif]

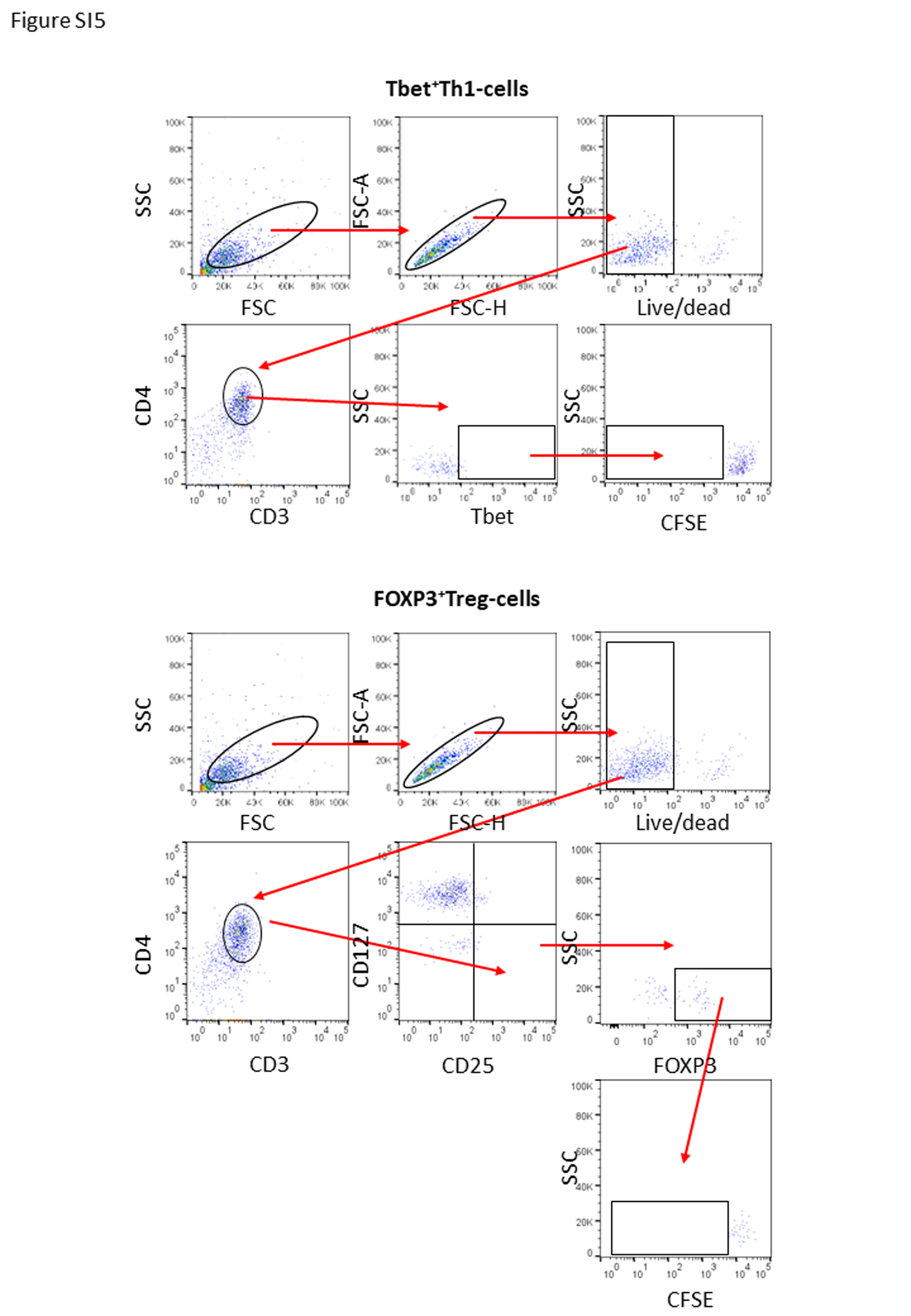

Supplement: Supplementary file 5 [file Image_5.tif]

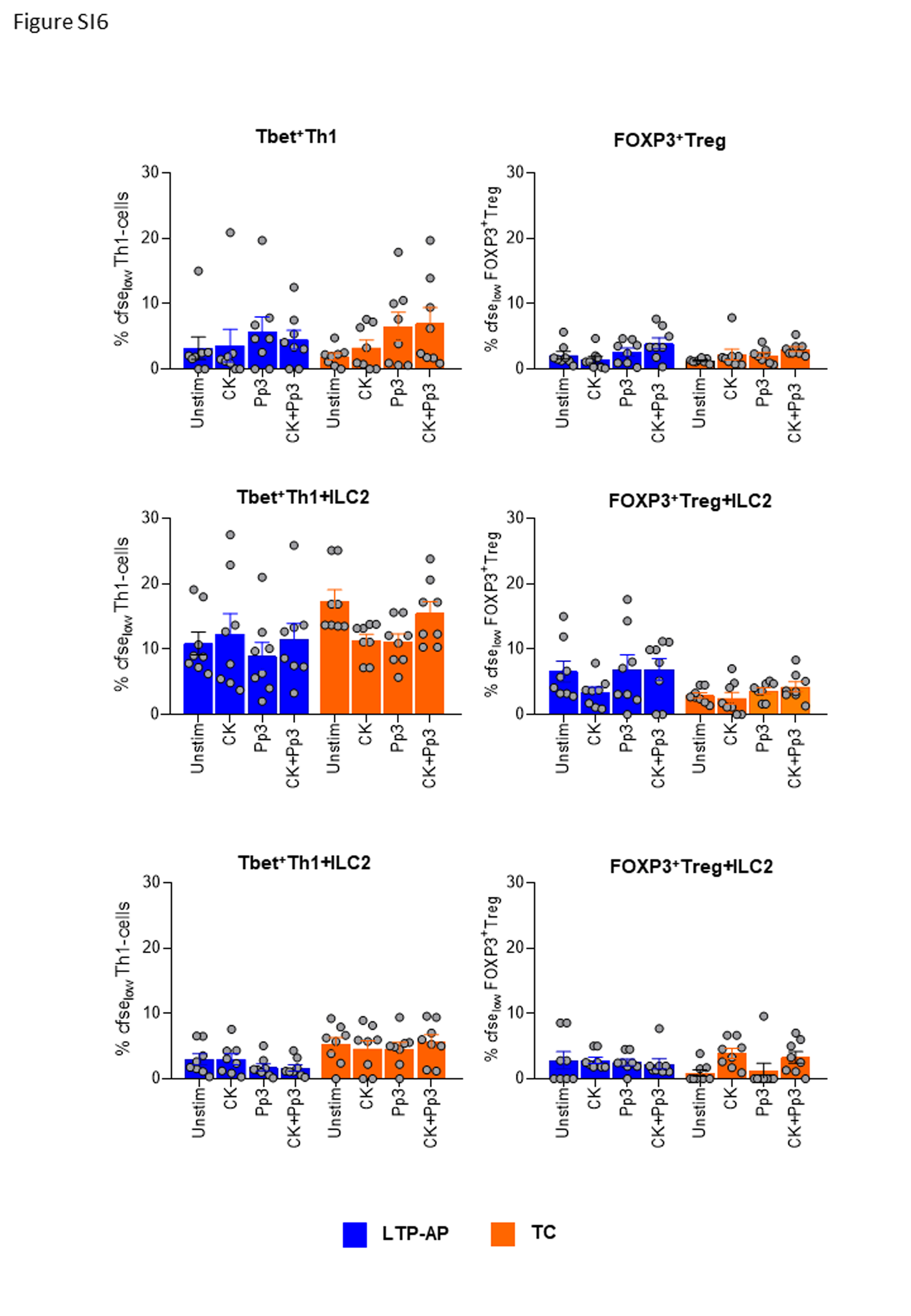

Supplement: Supplementary file 6 [file Image_6.tif]
