# Supplementary material for: Group 2 innate lymphoid cells are key in lipid transfer protein allergy pathogenesis
Source: Front Immunol. 2024 Apr 25;15:1385101. doi: 10.3389/fimmu.2024.1385101 (PMC11079275; doi:10.3389/fimmu.2024.1385101)
Supplement: Supplementary file 7 [file Table_1.docx]

**Supporting Information**

**Title:** Group 2 Innate lymphoid cells are key in lipid transfer protein (LTP) allergy pathogenesis.

**TABLE S1:** Monoclonal antibodies and probes used for flow cytometry.

| MoAbs/Probes | Fluorochrome | Clone | Vendor |
| --- | --- | --- | --- |
| Lineage Cocktail 1 | FITC | - | BD |
| CD15 | FITC | W6D3 | BD |
| CD123 | FTIC | 7G3 | BD |
| CD161 | PE/Cyanine7 | HP-3G10 | BioLegend |
| CD127 | PE | HIL-7R-M21 | BD |
| CRTH2 | PE | REA598 | Milteny Biotec |
| CD4 | APC | SK3 | BD |
| CD3 | Pacific Blue | UCHT1 | BioLegend |
| L/D stain kit | Near-IR | - | Thermo Fisher |
| GATA-3 | PerCP/Cyanine5.5 | 16E10A23 | BioLegend |
| T-bet | Brilliant Violet 605 | 4B10 | BioLegend |
| FOXP3 | PerCP/Cyanine5.5 | 236A/E7 | BD |
| CD83 | APC | HB15e | BD |
| CD86 | PerCP/Cyanine5.5 | 2331(fun-1) | BD |
| HLA-DR | Pacific Orange/PerCP | L234/clone | Exbio/BD |
| PD-L1 | FITC | MIH1 | BD |
| CFSE stain kit | - | - | Thermo Fisher |
|  |  |  |  |

MoAb: Monoclonal Antibody. CFSE: Carboxyfluorescein Succinimidyl Ester. Lineage Cocktail 1: CD3, CD14, CD16, CD19, CD20, CD56. L/D stain kit: Live/Dead stain kit.

**FIGURES AND FIGURE LEGENDS**

**Figure SI1.** **(A)** Frequency of viability of sorted LC2 in LTP-AP (N=12) and TC (N=12). **(B)** ILC2 absolute number in LTP-AP and TC and (C) flow cytometry analysis after the sorted ILC2. The bars with symbols represent mean and SEM of ILC2 viability and absolute number.

**Figure SI2**. Gating strategy using flow cytometry for co-stimulatory molecules expression by ILC2 and the histograms (smoothing style) of percentages of the different markers under the different experimental conditions from LTP-AP. Black line represents the histogram of the unstimulated cells, and horizontal lines represent the percentages of expression.

**Figure SI3**. **(A)** Gating strategy using flow cytometry to sort the T-cells, and T-cells confocal images after sorting. **(B)** Frequency (%) on sorted T-cells on alive PBMCs, absolute number and viability (%) of sorted T-cells in LTP-AP and TC (N=12, respectively). The bars with symbols represent mean and SEM of T-cells.

**Figure SI4**. Representative example of the strategies followed for the analysis of the CFSE low T-cell and Th2 response. The examples are taken from the co-cultures of T and ILC2 after 7 days of incubation in presence of Pru p 3 in LTP-AP, to show the results of the proliferation.

**Figure SI5**. Representative example of the strategies followed for the analysis of the CFSE low Tbet+Th1 and FOXP3^+^Treg response. The examples are taken from the co-cultures of T-cells and ILC2 after 7 days of incubation in presence of Pru p 3 in LTP-AP, to show the results of the proliferation.

**Figure SI6**. The bars with symbols represent mean and SEM of percentages of CFSE low expression in T-cell cultures alone, in ILC2/T-cell co-cultures and ILC2/T-cell co-cultures using Trans well (TW) plate for LTP-AP and TC (N=8, respectively) under different experimental conditions.
